# The Inhibitory Effect of Hafnium Oxide on Grain Growth in Yttrium Aluminum Garnet Composite Fiber

**DOI:** 10.3390/ma18235272

**Published:** 2025-11-21

**Authors:** Ke Gai, Qian Wang, Ketian Guan, Xiaohu Li, Weisen Liu, Yuan Li, Hongwei Zhao, Tong Zhao

**Affiliations:** 1 Key Laboratory of Science and Technology on High-Tech Polymer Materials, Institute of Chemistry, Chinese Academy of Sciences, Beijing 100190, China; 2 Science and Technology on Advanced Ceramic Fibers and Composites Laboratory, National University of Defense Technology, Changsha 410073, China; 3 Beijing National Laboratory for Molecular Sciences, Beijing 100190, China; 4 Shanghai Rongrong New Materials Technology Co., Ltd., Shanghai 201413, China; 5 University of Chinese Academy of Sciences, Beijing 100049, China

**Keywords:** YAG, HfO_2_, grain size, ceramic fiber

## Abstract

Yttrium aluminum garnet (YAG, Y_3_Al_5_O_12_) fibers are promising materials for high-power lasers and high-temperature structural materials, and it is anticipated that the improvement in the stability of grain size would extend their service life at high temperatures. In this work, YAG-HfO_2_ composite ceramic fibers were obtained by the solution blow spinning of YAG-HfO_2_ composite precursor and sintering in steam. The effect of HfO_2_ on the crystal phase transition and grain growth of YAG-HfO_2_ fibers was further studied by in situ X-ray Diffraction (XRD), Scanning Electron Microscope (SEM), and Transmission Electron Microscope (TEM). The results show that the HfO_2_ addition increased the crystallization temperature of the YAG phase from 900 °C to 950 °C and reduced the crystal size at 1400 °C from 41.9 nm to 31.8 nm. The HfO_2_ grains were distributed at the boundary of YAG grains, which enabled the fiber to maintain its dense structure and uniform grain size even at 1500 °C, exhibiting excellent high-temperature grain size stability of composite fibers.

## 1. Introduction

Yttrium aluminum garnet (YAG, Y_3_Al_5_O_12_) ceramic fibers have emerged as critical structural materials for next-generation high-temperature applications, particularly in aerospace propulsion systems [1,2,3,4]. Their appeal lies in an exceptional combination of thermophysical properties, including high melting point (>1970 °C), superior creep resistance, outstanding phase stability, and low radiation-induced swelling [5,6,7,8]. The grain size is of critical importance for oxide ceramics in high-temperature applications. Polycrystalline YAG exhibits three times higher grain size stability than polycrystalline alumina with the same grain size at 1400 °C [9]. Thus, YAG ceramic fiber is regarded as a superior alternative for applications under high-temperature conditions [10,11,12].

However, when subjected to prolonged thermal exposure at service temperatures for long periods, single-phase YAG fibers undergo grain coarsening, which is a common problem of single-phase polycrystalline ceramic fibers [13,14]. For ceramic fibers, a fine-grained microstructure significantly improves multiple properties such as strength, toughness, thermal shock resistance, light transmittance, and thermal stability [15,16]. To solve this issue, refractory oxide dopants have been strategically incorporated into single-phase polycrystalline ceramic fibers to inhibit grain growth.

Many efforts have been devoted to minimizing the grain size of YAG ceramic fibers. Due to the poor grain size stability of Al_2_O_3_ at high temperatures, many other oxides have been considered the candidates to inhibit the YAG fiber’s grain growth. The Zr element was introduced into YAG to obtain ceramic fibers enhanced by a second phase [17]. However, instead of forming a second ZrO_2_ phase at the YAG grain boundaries as anticipated, the Zr element was dispersed within the YAG grains. This solid solution reduced the crystallization activation energy of the Zr-doped YAG fibers to 59.69 kJ/mol, a value lower than that reported for undoped YAG. Consequently, this lower activation energy facilitated YAG grain growth, ultimately limiting the high-temperature application of these fibers.

In contrast, Mg element was added to YAG to create composite ceramic fibers [18]. After heating at 1600 °C for 2 h and 8 h, respectively, the generated MAS (MgAl_2_O_4_) grains were distributed at the boundaries of the YAG grains, thereby inhibiting the average grain size of the fibers to 1.3 µm and 1.9 µm, respectively. However, this study did not provide a direct comparison with the grain growth of undoped YAG fibers under identical conditions, so the inhibitory effect was not investigated. And controlling grain sizes below 1 micrometer or even sub-micron is still a crucial challenge.

As a typical refractory oxide, hafnium oxide (HfO_2_) is an ideal candidate component for grain growth inhibitors due to its extremely high melting point (~2800 °C) and excellent thermodynamic stability [19,20]. The efficacy of this approach is evidenced by its successful incorporation into Al_2_O_3_ and Al_2_O_3_-mullite fibers, where it significantly improved grain size stability [21,22,23]. In our previous work, the Al_2_O_3_-mullite fibers without HfO_2_ underwent severe grain coarsening after only 0.5 h at 1500 °C. In contrast, fibers with 50 wt.% HfO_2_ maintained a fine, sub-200 nm average grain size under the same conditions, with HfO_2_ particles located at the grain boundaries effectively pinning the microstructure. Although the YAG ceramic fibers reported in our previous work showed a high-temperature resistance at 1200 °C, their grain size increased quickly when the temperature was elevated above 1300 °C [24]. Therefore, introducing HfO_2_ into YAG fiber presents a promising strategy to improve its grain size stability at higher temperatures.

In this work, a YAG-HfO_2_ composite precursor was synthesized, and the ceramic fibers were prepared. The thermal behavior and chemical structure of the YAG-HfO_2_ fibers were analyzed using Simultaneous Thermal Analyzer (TG-DSC) and Fourier Transform Infrared (FT-IR). The effects of HfO_2_ on the crystal phase transition and grain growth of the YAG phase were compared by in situ XRD, SEM, and TEM. The effect of HfO_2_ grains distributed at the boundary of YAG grains on the grain growth even at 1500 °C was investigated, and the results show that the composite fibers’ high-temperature stability improved significantly.

## 2. Experimental Section

### 2.1. Materials

Aluminum isopropoxide (C_9_H_21_AlO_3_, AR, 99.8%) was supplied by Shanghai Haosheng Chemical Science-Technology Co., Ltd. (Shanghai, China). Acetylacetone (C_5_H_8_O_2_, CP, 99.5%), Isopropanol (C_3_H_8_O, CP, 99.5%), Ethanol Alcohol (C_2_H_5_OH, CP, 99.5%), Ethylene glycol monoethyl ether (C_4_H_10_O_2_, CP, 99.5%) and Deionized water (H_2_O) was supplied by Modern Oriental (Beijing) Technology Development Co., Ltd. (Beijing, China). Polyvinyl pyrrolidone ((C_6_H_9_NO)_n_, GR, average mol wt.% 1,300,000) and Yttrium acetylacetonate (C_15_H_23_O_7_Y, REO, 99.9%) was offered by Shanghai Macklin Biochemical Co., Ltd. (Shanghai, China). Hafnium alkoxide was prepared according to the literature [25].

### 2.2. Preparation of YAG-HfO_2_ Precursor and Fiber

**Figure 1:**
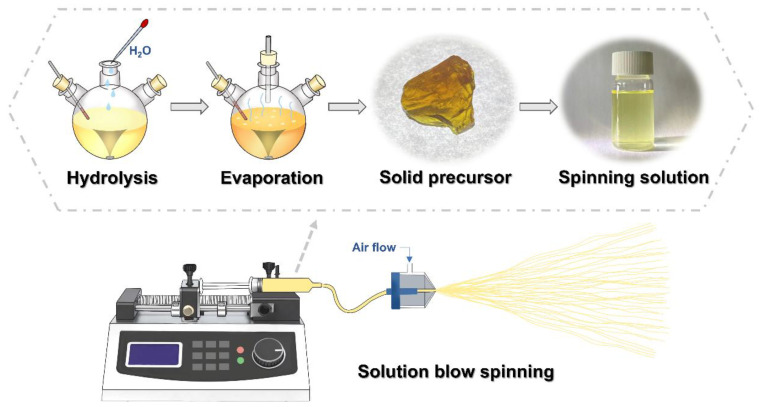
The preparation process of the YAG-HfO_2_ precursor and fiber.

The preparation process of the YAG-HfO_2_ precursor and fiber is shown in Figure 1. The masses of materials were calculated from the target Y:Al:Hf cationic molar ratio of 3:5:0.09, which corresponds to a final composition of Y_3_Al_5_O_12_ with 10 wt.% HfO_2_. Firstly, 100 g of aluminum isopropoxide was dissolved in 150 g of isopropanol at 80 °C. Next, 110 g of yttrium acetylacetonate was added dropwise to the solution, followed by an addition of 8 g of water and 20 g of isopropanol for the metallic compound’s hydrolysis process. After the addition of 2 g of acetylacetone to 22 g of hafnium alkoxide at 80 °C, 0.6 g of water and 2 g of isopropanol were added to the mixture. Subsequently, the ethylene glycol ether was added after mixing those two solutions, and the solvent mixture was removed in vacuo at 140 °C to acquire the YAG-HfO_2_ solid precursor, in which the HfO_2_ content was 10 wt.%.

YAG-HfO_2_ fiber was produced by the solution blow spinning of the YAG-HfO_2_ solid precursor and polyvinyl pyrrolidone dissolved in ethanol with a specific viscosity. A spinneret with an inner diameter of 0.3 mm, a solution feed rate of 1 mL/min, and a gas pressure of 0.4 MPa was used in the solution-blowing process to prepare green fiber. Then the green fiber was sintered to 800 °C in steam with a heating rate of 0.5 K/min.

### 2.3. Characterization

The thermal behavior of the YAG-HfO_2_ green fiber was examined using TG-DSC (TGA/DSC3+, Mettler Toledo, Greifensee, Switzerland) with a heating rate of 10 K/min in airflow. FT-IR spectra of YAG-HfO_2_ fiber were carried out with FT-IR spectroscopy (TENSOR27, Bruker, Mannheim, Germany) between 4000 and 400 cm^−1^. The crystal phase transition during heating at 100 °C/min was investigated by in situ X-ray diffraction using a Bruker D8 ADVANCE diffractometer (Bruker, Mannheim, Germany) equipped with an Anton Paar HTK 2000N high-temperature chamber (Anton Paar, Graz, Austria). Measurements were conducted under Cu Kα radiation with a 2θ scanning range from 10° to 80° at a rate of 8 °/min, where an isothermal hold of 1 min was applied at each target temperature before scanning to ensure thermal equilibrium. The crystal size was subsequently calculated from the acquired data using Jade 6. The microstructure of YAG-HfO_2_ fiber was observed by SEM (S-8020, Hitachi, Tokyo, Japan). Elemental analysis was conducted using energy-dispersive X-ray spectroscopy (EDS) with an Oxford detector attached to the SEM. Both SEM imaging and EDS mapping were conducted at an accelerating voltage of 10 kV and a working distance of 8~10 mm, and the EDS elemental maps were acquired with a dwell time of 30–60 s. The transmission electron microscope (TEM) and high-resolution transmission electron microscope (HR-TEM) images were obtained using a TEM (JEM-F200, JEOL, Tokyo, Japan) operated at 200 kV. The energy dispersive spectroscopy (EDS) linked to the TEM was used to confirm the distribution of elements of the fibers. Digital Micrograph 3.7 was used to measure the lattice spacing from HR-TEM images.

## 3. Results and Discussion

**Figure 2:**
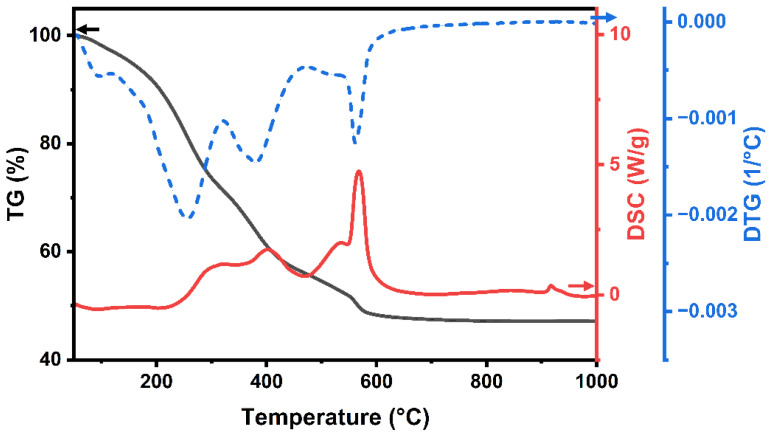
TG-DSC-DTG curves of YAG-HfO_2_ green fiber.

The decomposition behavior of YAG-HfO_2_ green fiber prepared by solution spinning was examined by TG-DSC. As shown in Figure 2, the organic component was fully pyrolyzed when the residual weight of the green fibers at 1000 °C was close to 50% in the TG curve. It can be seen in the Derivative Thermogravimetry (DTG) curve that the green fibers lost just 3% of their weight when heated from room temperature to 120 °C, which might be caused by the volatilization of residual solvents. The decomposition and removal of the acetylacetone ligands accounted for the 26% and 15% weight loss of the green fibers in the 120–320 °C and 320–470 °C ranges, respectively [2,21]. This thermal decomposition process involved simultaneous bond scission and the evolution of gaseous products, such as CO_2_, H_2_O, and acetylacetone fragments. At 470–800 °C, the weight loss of green fibers was 9%, which was mostly a result of the further decomposition of remaining organic groups and the slow oxidation of residual carbon. The DSC curve shows comparable exothermic peaks for each of the thermal decomposition stages. Additionally, a small exothermic peak was observed above 900 °C. This peak is conclusively identified as the crystallization of the YAG phase, which is directly evidenced by the in situ XRD results discussed below.

**Figure 3:**
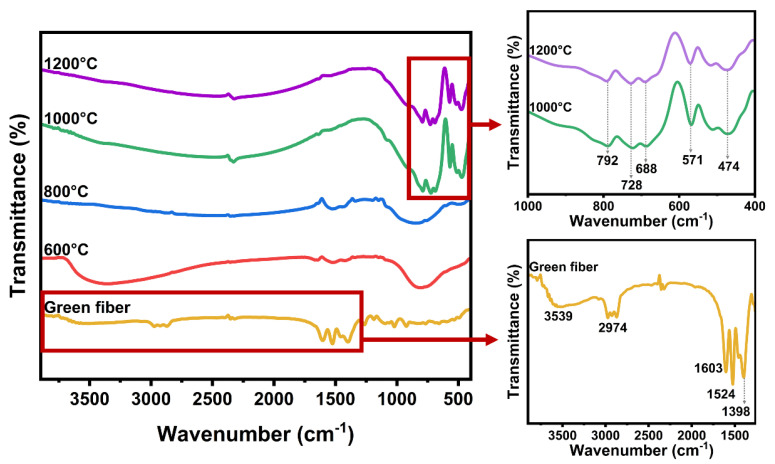
FT-IR spectra of YAG-HfO_2_ green fiber and the fiber sintered at different temperatures.

The composition of the YAG-HfO_2_ green fiber and fiber sintered at different temperatures was investigated by FT-IR spectra (Figure 3). The band at 3597 cm^−1^ corresponded to the stretching vibration of -OH, which may be caused by the residual isopropyl groups. Isopropyl was identified by absorption of C-H at 2974 cm^−1^. Acetylacetone ligands were detected according to the strong absorption at 1603 cm^−1^, 1524 cm^−1^, and 1398 cm^−1^, belonging to the stretching vibration of C=O, C=C, and C-CH_3_, respectively [26,27]. As the sintered temperature increased to 600 °C and 800 °C, the vibrations associated with solvents and acetylacetone ligands gradually disappeared, indicating the removal of organic components. When the sample was sintered at 1000 °C and 1200 °C, the absorption peak representing the inorganic substance appeared. The bands at 792 cm^−1^ and 688 cm^−1^ were commonly assigned to the Al-O vibrations. The bands at about 728 cm^−1^ and 474 cm^−1^ were commonly assigned to Y-O stretching vibrations. The bands at 571 cm^−1^ were related to the stretching vibration of Al-O-Y [28,29,30]. The above absorption peaks of Al-O, Y-O, and Al-O-Y indicated the appearance of the YAG phase in the sample, which was consistent with the DSC curve, and the YAG composite fibers underwent densification and microstructural evolution at this temperature, achieving the transformation from organic fibers into ceramic fibers.

**Figure 4:**
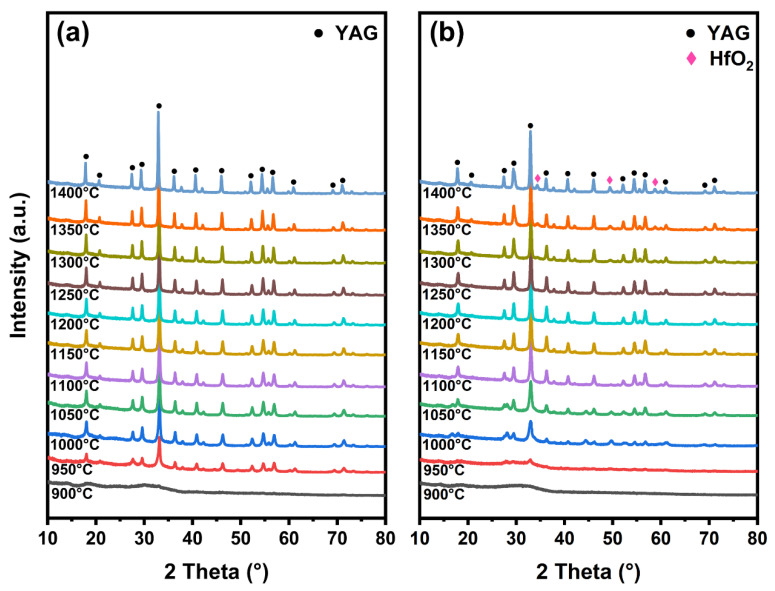
In situ XRD patterns of YAG fibers (**a**) and YAG-HfO_2_ fibers (**b**) heating from 900 °C to 1400 °C.

The crystal phase transition process heating from 900 °C to 1400 °C was investigated by in situ XRD of the YAG and YAG-HfO_2_ inorganic fibers obtained at 800 °C, and the effect of HfO_2_ on the grain growth of both fibers with rising temperature was further investigated. As shown in Figure 4a, for YAG fibers, weak diffraction peaks of the YAG phase (PDF#79-1892) were observed at 900 °C, and as the temperature increased, the intensity of the YAG diffraction peak significantly enhanced. The diffraction peaks at 18.07°, 22.77°, 29.74°, 33.32°, 36.62°, 41.15°, 46.60°, 52.78°, 54.39°, and 56.65° represented the (2 1 1), (3 2 1), (4 0 0), (4 2 0), (4 2 2), (5 2 1), (5 3 2), (4 4 4), (6 4 0), and (6 4 2) crystal planes of YAG, respectively, with no intermediate phases detected. For the YAG-HfO_2_ fibers in Figure 4b, the weak YAG diffraction peaks emerged at 950 °C, which was in good qualitative agreement with the exothermic peak in the DSC curve. This delayed crystallization temperature was ascribed to the Hf elements’ dispersion in the fibers, which hindered the nucleation of the YAG phase. In the amorphous state of fibers, the uniformly dispersed Hf elements hindered the diffusion of Y, Al, and O cations required to form stable YAG critical nuclei, which increased the kinetic barrier for nucleation, thereby raising the crystallization temperature from 900 °C to 950 °C. The diffraction peaks of 35.06°, 50.42°, and 59.93° corresponded to the (2 0 0), (2 2 0), and (3 1 1) crystal planes of *c-*HfO_2_ (PDF#70-2831) appeared at 1250 °C. Moreover, the consistently lower diffraction peak intensities for YAG-HfO_2_ fibers confirm the sustained inhibitory effect of HfO_2_ grains on YAG crystallinity and crystal size throughout the heating process.

**Figure 5:**
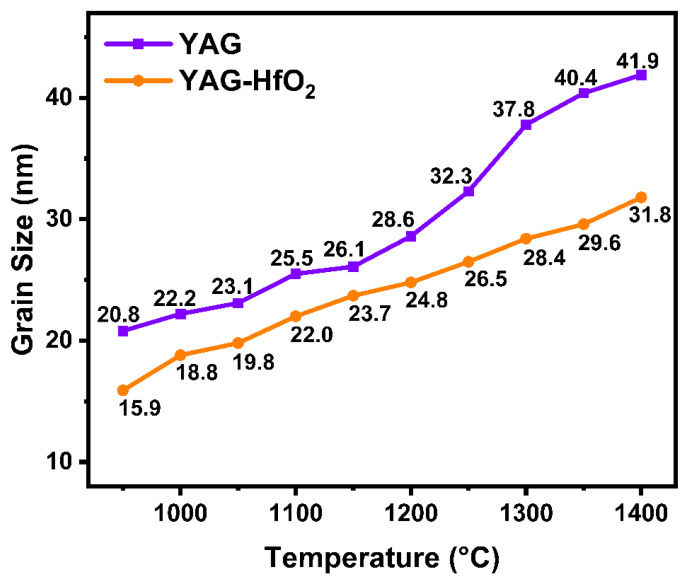
Calculated average crystal size of YAG grains from in situ XRD data of YAG and YAG-HfO_2_ fiber heating from 950 °C to 1400 °C.

The average crystal size of YAG grains at different temperatures during the heating process was calculated by the Debye–Scherrer formula to further investigate the effect of HfO_2_ on the grain growth of YAG fiber. As shown in Figure 5, the crystal size of the YAG fiber was 20.8 nm at 950 °C, and then slowly increased as the temperature rose to 1200 °C. Above 1250 °C, accelerated grain growth was observed, with the size reaching 41.9 nm at 1400 °C. In contrast, the crystal size of YAG-HfO_2_ fiber was consistently finer, measuring 15.9 nm at 950 °C. The crystal size of the YAG-HfO_2_ fiber increased gradually as the temperature rose, and it continued to be smaller than YAG fiber, reaching 31.8 nm at 1400 °C. This represents a 24% reduction in final crystal size at 1400 °C due to HfO_2_ addition. The increase in crystal size with increasing temperature, as observed in both YAG and YAG-HfO_2_ composite fibers, is a classic phenomenon in polycrystalline materials driven by the reduction in total interfacial energy within the system, and it is evident that the HfO_2_ component added can effectively prevent YAG grains from growing by the Zener pinning mechanism, enhancing the YAG-HfO_2_ fiber’s resistance to high temperatures.

**Figure 6:**
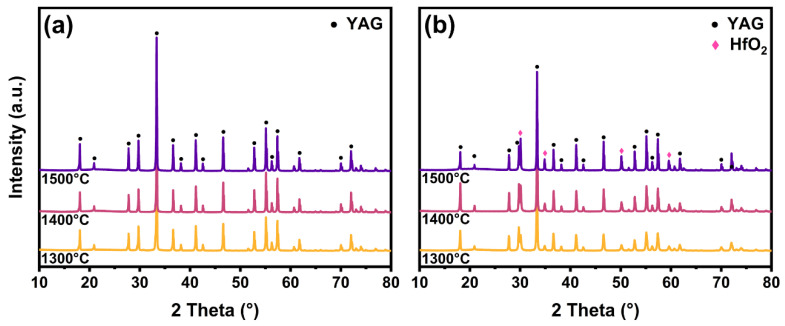
XRD patterns of YAG fibers (**a**) and YAG-HfO_2_ fibers (**b**) sintered at 1300 °C, 1400 °C, and 1500 °C for 10 min, respectively.

To further study the effect of HfO_2_ on the microstructure of YAG fiber at high temperatures, the YAG and YAG-HfO_2_ fibers were sintered at 1300 °C, 1400 °C, and 1500 °C for 10 min, respectively, and were compared by XRD, SEM, and TEM. As shown in Figure 6, the XRD patterns collected at room temperature from fibers confirm that both the YAG and YAG-HfO_2_ fibers were fully crystallized under the applied sintering conditions. Furthermore, distinct diffraction peaks corresponding to HfO_2_ were clearly identified in the composite fibers, confirming the successful incorporation of the second phase. The microstructural evolution and grain growth were subsequently investigated by SEM and TEM.

**Figure 7:**
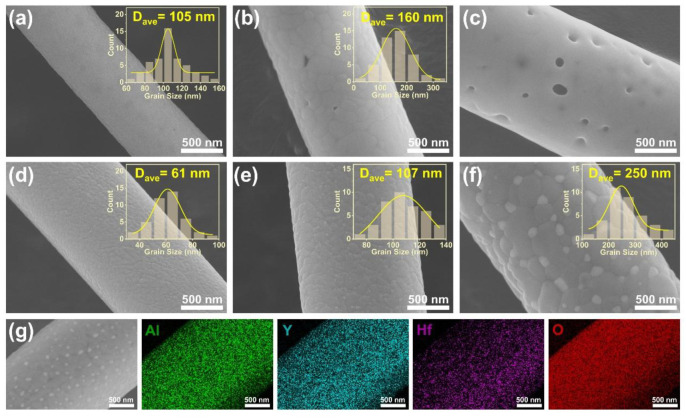
SEM images of YAG fiber (**a**–**c**) and YAG-HfO_2_ fiber (**d**–**f**) sintered at 1300 °C, 1400 °C, and 1500 °C for 10 min, respectively, and the EDS-Mapping images of YAG-HfO_2_ fiber sintered at 1500 °C for 10 min (**g**).

The SEM images in Figure 7 show the microstructural evolution of both fibers at high temperatures. At 1300 °C (Figure 7a), the YAG fiber had a dense surface with uniform grain sizes of ~105 nm. When the temperature rose to 1400 °C (Figure 7b), significant coarsening occurred, with the average grain size on the surface of the fiber increasing to ~160 nm, and a less dense structure with holes emerged as a result of the larger grain size. At 1500 °C (Figure 7c), the YAG grains underwent tremendous growth, and grain boundaries could no longer be observed at the same scale, making statistical grain size analysis unreliable at this scale. Additionally, numerous holes were generated within and on the surface of the fiber. In contrast, for the YAG-HfO_2_ fiber, the crystal structures after sintering at temperatures of 1300 °C, 1400 °C, and 1500 °C were all dense (Figure 7d–f). The grain sizes were statistically smaller and more uniform, measuring ~61 nm at 1300 °C, ~107 nm at 1400 °C, and ~250 nm at 1500 °C. This is significantly finer than the ~800 nm grains reported for pure YAG fibers under the same temperatures in the literature [6]. This suppression of grain growth is attributed to the Zener pinning mechanism. As shown in Figure 7g, direct microstructural evidence confirms that the finely dispersed HfO_2_ particles were located at the YAG grain boundaries. While a similar pinning mechanism was reported in Mg-doped YAG [18], the effect is more pronounced in our work due to the higher content of HfO_2_ particles. Benefiting from the growth inhibition effect of HfO_2_ grains dispersed at the grain boundaries of YAG grains, YAG-HfO_2_ fibers can still retain a dense structure even at 1500 °C.

**Figure 8:**
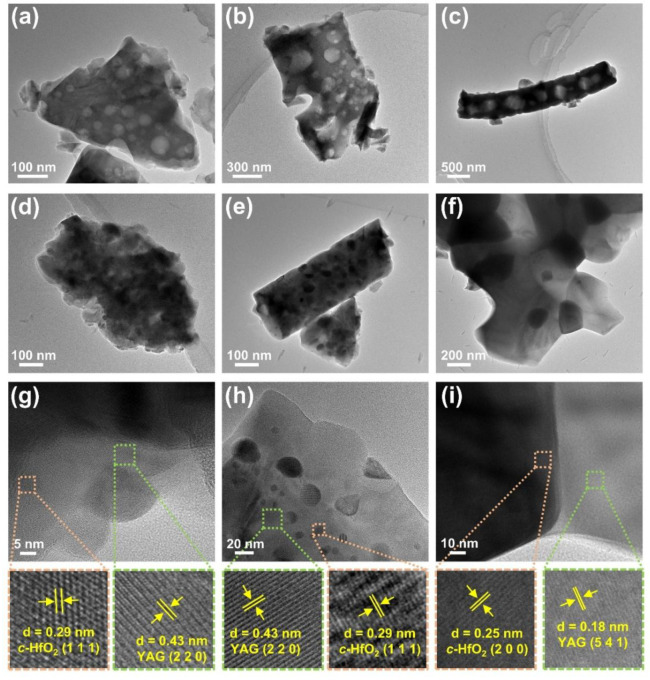
TEM images of YAG fiber (**a**–**c**) and YAG-HfO_2_ fiber (**d**–**f**) sintered at 1300 °C, 1400 °C, and 1500 °C for 10 min, respectively, and the HR-TEM images of YAG-HfO_2_ fiber sintered at 1300 °C, 1400 °C, and 1500 °C for 10 min (**g**–**i**), respectively.

**Figure 9:**
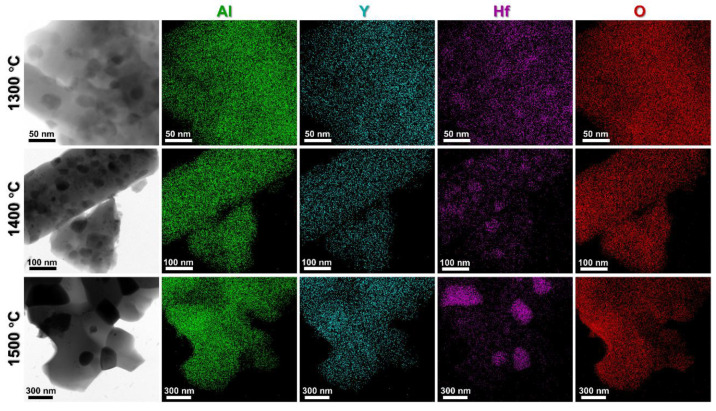
EDS-Mapping images of YAG-HfO_2_ fiber sintered at 1300 °C, 1400 °C, and 1500 °C for 10 min.

TEM analysis was performed to further investigate the microstructure of the YAG and YAG-HfO_2_ fibers sintered at different temperatures. For the YAG fiber at 1300 °C (Figure 8a), the internal structure was found to contain numerous pores with sizes ranging from 20 to 80 nm. With the temperature increased to 1400 °C (Figure 8b), the pores within the fibers were found to enlarge and become more irregular in size, indicating a further increase in microstructural porosity. At 1500 °C (Figure 8c), the pores in the fibers were found to approach ~500 nm, and the grain boundaries within the fibers could no longer be clearly distinguished. In contrast, the YAG-HfO_2_ fiber (Figure 8d–f) was characterized by a dense microstructure at all temperatures, which was consistent with the SEM observations. The porosity in YAG fibers was initiated by rapid abnormal grain growth, as a consequence of the kinetic imbalance between boundary migration and densification. The introduction of HfO_2_ particles inhibited this process through a Zener pinning mechanism, which restricted abnormal grain boundary migration and thereby prevented the formation of pores. This mechanism was directly evidenced by our microstructural observations, where black and gray grains were observed in the YAG-HfO_2_ fibers and characterized by HR-TEM (Figure 8g–i). The lattice spacing of the small black grains in YAG-HfO_2_ fibers sintering at 1300 °C was measured to be 0.29 nm, corresponding to the (1 1 1) crystal plane of *c*-HfO_2_, while the larger gray grains exhibited a lattice spacing of 0.43 nm, consistent with the YAG crystal plane (2 2 0). As the temperature further increased, the lattice spacing of *c*-HfO_2_ and YAG was also observed and further confirmed. The element distribution of both Al, Y, Hf, and O was validated by EDS-Mapping in Figure 9. From this, the grain distribution of HfO_2_ and YAG was further confirmed. It can be seen that HfO_2_ grains were distributed at the grain boundaries of YAG, thereby hindering abnormal grain growth and the formation of pores, enhancing the microstructural stability of the fiber at high temperatures.

## 4. Conclusions

In this work, HfO_2_ was introduced into YAG ceramic fiber as a grain growth inhibitor, successfully fabricating a novel YAG-HfO_2_ composite fiber with enhanced high-temperature stability. The effect of HfO_2_ on the phase transition and microstructural evolution was systematically investigated via in situ XRD, SEM, and TEM. The main findings are conclusively summarized as follows:

1. Compared with pure YAG fiber without HfO_2_, the introduction of HfO_2_ delayed the crystal phase transformation of the YAG-HfO_2_ fibers, increasing the crystallization temperature of the YAG phase from 900 °C to 950 °C.
2. The grain growth in YAG-HfO_2_ fibers was consistently suppressed, resulting in a steadier and finer microstructure. The crystal size of YAG-HfO_2_ fiber was only 31.8 nm at 1400 °C, notably smaller than the 41.9 nm in pure YAG fibers.
3. The suppression mechanism was identified as Zener pinning, where HfO_2_ particles located at YAG grain boundaries effectively impeded grain coarsening. YAG-HfO_2_ fibers had an average grain size of ~250 nm at 1500 °C, demonstrating exceptional microstructural stability at high temperatures.

Thus, the successful fabrication of YAG-HfO_2_ fiber offers a promising approach to improving the high-temperature stability and fine-tuning grain size of oxide ceramic fiber, creating novel prospects for multicomponent ceramic and aerospace applications.

## Data Availability

The original contributions presented in this study are included in the article. Further inquiries can be directed to the corresponding author.
